# Woody Species Composition, Plant Communities, and Environmental Determinants in Gennemar Dry Afromontane Forest, Southern Ethiopia

**DOI:** 10.1155/2022/7970435

**Published:** 2022-06-22

**Authors:** Shemsu Ahmed, Debissa Lemessa, Abera Seyum

**Affiliations:** ^1^Forest and Rangeland Plant Biodiversity Directorate, Ethiopian Biodiversity Institute, Addis Ababa, Ethiopia; ^2^Addis Ababa University, Addis Ababa, Ethiopia

## Abstract

Dry Afromontane forests in Ethiopia are vital for the conservation of plant diversity and climate change mitigation. However, these forest resources are rapidly degrading and shrinking, necessitating empirical scientific investigations to ensure their successful conservation and long-term management. As a result, this study was conducted to evaluate the composition, plant communities, and environmental determinants of woody species in the Gennemar dry Afromontane forest of southern Ethiopia. Environmental variables such as altitude, aspect, and geographical location were recorded from 46 plots of 20 × 20 m for trees and 92 subplots of 10 × 10 m for shrubs were laid along 10 transect lines. Vegetation structure, diversity, vegetation classification, importance value index (IVI) and correlation with environmental parameters were analyzed. A total of 55 woody species belonging to 51 genera and 34 families were identified. Among the species identified, *Jasminum stans, Maytenus addat,* and *Pittosporum abyssinicum* were endemic to Ethiopia. Celastraceae (with 659 individuals) was the most dominant family, followed by Cupressaceae (268 individuals) and Myrsinaceae (222 individuals). Four plant communities were identified: *Syzygium guineense-Mystroxylon aethiopicum, Maytenus arbutifolia-Podocarpus falcatus, Myrsine africana-Erica arborea,* and *Juniperus procera-Carissa spinarum*. *Juniperus procera*, *Podocarpus falcatus*, and *Maytenus arbutifolia* were species with the highest IVI, while *Maesa lanceolata, Rhamnus prinoides,* and *Gnidia glauca* had the lowest. The DBH class distribution shows an *inverted J-shaped distribution.* As DBH increases, the number of individuals decreases in the higher DBH class. The distribution of plant communities and the composition of the species depend on altitude and topographic aspects. The study found that the dry Afromontane Forest is rich in species and that it should be prioritized for conservation to protect endemic and native species. Decisive elements such as the type of species, altitude, and topographic aspects must be considered for forestry activities.

## 1. Introduction

Ethiopia is a mountainous country with striking contrasts: rugged mountains, flat plateaus, deep gorges and river valleys, and rolling plains. Its elevation varies from 110 meters below sea level in the Dallol depression to 4620 meters at the highest peak of the Ras Dejen [1]. The proximity of the Equator on Ethiopia's southern border and the complexity of the topography are the two key topographic variables that govern the country's climate, resulting in seasonal variability, annual precipitation, and climatic conditions [2–4]. Topographic factors, such as soil moisture, chemical qualities (nitrogen, potassium), physical (drainage, porosity, etc.), and other characteristics, all play a role in the distribution and diversity of plant species [5–7]. Ethiopia is one of the 25 biodiversity rich countries of the world, and about 6500 species of vascular plants has been estimated [8]. However, this plant genetic resource of Ethiopia has been reduced due to deforestation for agricultural land, timber products, fuelwood, settlement, and other anthropogenic effects [9–11]. Forest degradation causes not only depletion of the forest resources but also leads to loss of other fauna and flora as it is home for these fauna and flora, soil degradation, soil erosion, reduction in underground water and annual precipitation, and other natural resource depletion [12].

The Afromontane forest covers more than half of the total land area in Ethiopia's highlands, with dry evergreen Afromontane forests accounting for more than half of that [13]. This dry Afromontane forest is found in Ethiopia's northern, northwestern, central, southern, southeastern, and southwestern regions at elevations ranging from 1800 to 3000 meters above sea level [4]. *Juniperus–Podocarpus* forests or primarily Podocarpus forests, both with broad-leaved species, make up these dry Afromontane forests [14]. Despite this, the dry Afromontane forest resource of Ethiopia is currently under significant threat, mainly due to fast population increase as natural products and lands are the mainstays of the population's livelihood, and demand for both is steadily increasing [13, 15].

Analysis of species composition provides natural sustainability for all living forms, as well as the conservation of species, ecosystem, communities, and habitats of an organism for upcoming generations and proper use of genetic resources [16]. Environmental factors such as altitude, slope, and aspect have a significant impact on the diversity and richness of woody species [17]. However, the effect of these environmental factors was poorly understood [18, 19]. Gennemar natural forest is a dry evergreen Afromontane forest that is located in the Gurage Zone in Southern Ethiopia. This forest is one of the dominant dry Afromontane forests and is enclosed by agricultural land. It serves as the home to indigenous plant species, mammals, and birds. The effect of altitude and topographic aspect on species and plant community distribution in the Gennemar natural forest has not been investigated so far. Therefore, this study was carried out with the objective of determining the woody species composition and its environmental determinants in the natural forest.

## 2. Materials and Method

### 2.1. Description of the Study Area

**Figure 1:**
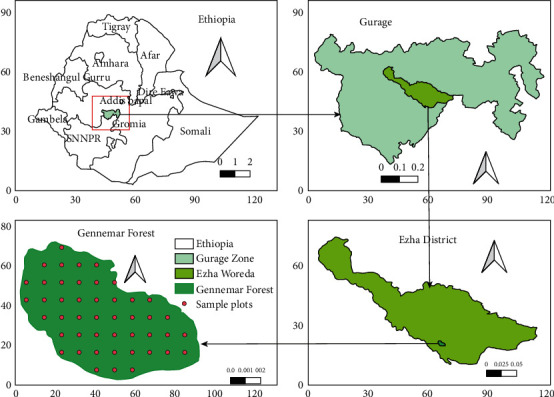
Map of study area in relation to the maps of the district, zone and Ethopia.

The study was conducted at Gennemar natural forest of Ezha district (*Woreda,*Figure 1) which is located in the Gurage Zone of the South Nations and Nationalities People Region. The geographical location of the district lies between 8^o^03′–8^o^16′ N latitude and 37^o^50′–38^o^12′E longitude. Gennemar natural forest is one of the Dry Afromontane Forests found in Ezha district, with an area coverage of 85.8 ha [20]. The district receives a range of annual rainfall between 900 and 1600 mm and a range of annual temperatures of 5−38°C [21]. The study area is situated 200 km southwest of Addis Ababa.

### 2.2. Sampling Design

A systematic sampling procedure was applied to collect vegetation data from the study forest. Transects and sample plots were laid out based on the total area of the forest using QGIS (version 3.6). Ten transects and 46 main sample plots of 20 m × 20 m were laid horizontally at 125 m intervals. Here, the length of each transect varies based on the extent of the forest edges and ranges between 0.43–0.97 km. To collect data on shrubs, ninety-two subplots of size 10 m × 10 m are located at the two corners of the sample plots.

### 2.3. Data Collection

All woody species found in the sample plots were recorded and given a local name. Outside of the sample plots but within the forest, they were recorded as present but not included in the vegetation data analysis. The species list and number of individuals for each species in the sample plot were recorded. Trees and shrubs whose DBH (diameter at breast height) are ≥5 cm were measured using a caliper. A Garmin GPS (±3 m) was used to navigate to sample plots and record the elevation of the sample plots. The topographic aspects of the sample plots were identified using the Silva Compass. The plant specimens were collected for all woody species, pressed using a plant press, and transported to the herbarium of the Ethiopian Biodiversity Institute. After the plant specimens collected from the field were ventilated and dried well in the sunlight, they were identified to the species level, mounted, labeled, and conserved in the herbarium of the Ethiopian biodiversity institute.

### 2.4. Data Analysis

#### 2.4.1. Species Richness and Diversity

The diversity of woody species, the classification of plant community types, and their relation to altitude and topographic aspects were determined by multivariate analysis using the vegan package R version 4.0.2 [22]. Hierarchical cluster analysis techniques using similarity ratios were used to classify the types of plant communities. The Shannon–Wiener diversity index (*H*′) which is the most commonly used) was applied to estimate species diversity based on species richness and evenness [23].

(1) $$\begin{array}{c} H^{\prime }=\sum_{i=1}^{s}Pi\;\ln\left(Pi\right). \end{array}$$

where *H*′ is Shannon–wiener index, *pi* is the proportion of the total abundance of the community represented by the *i*-th species, ln (*pi*) is the natural log of *pi*, *S* = number of species encountered, and Σ = sum of species 1 to species *S*.

#### 2.4.2. Plant Community and Environmental Factors

To investigate vegetation data and environmental factors, the vegan package's Adonis function was used, which fits linear models to distance matrices and uses 999 permutation tests using Pseudo F-ratios. The Adonis function implements a multivariate analysis of variances (MANOVA) using distance matrices and draws a PCA plot with only those environmental variables that show significant effects (in our case *P* < 0.01) on the composition of species of communities [24]. The relationship between environmental factors and specific communities depends on the relative distance between them [22].

#### 2.4.3. Importance Value Index

The importance value index (IVI) was used to compare the overall dominance and ecological significance of species using the following formula, which integrates data from three factors (relative density, relative frequency, and relative dominance) [25]:

(2) $$\begin{array}{c} \text{IVI}=\text{relative density}+\text{relative frequency}+\text{relative dominance}. \end{array}$$

Relative density is the study of the numerical strength of a species in relation to the total number of individuals of all the species and is calculated as

(3) $$\begin{array}{c} \text{relative density}=\frac{\text{number of individuals of species}}{\text{number of individual of all species}}*100. \end{array}$$

Relative frequency is the degree of individual species distribution in a given area in relation to the total number of species that occurred.

(4) $$\begin{array}{c} \text{relative frequency}=\frac{\text{number of occurrence of the species}}{\text{number of occurrence of all the species}}*100. \end{array}$$

Relative dominance is the coverage value of a species with respect to the sum of coverage of the rest of the species in the area.

(5) $$\begin{array}{c} BA=0.0000785*{\left(DBH\right)}^{2}, \end{array}$$

where BA = basal area and DBH = diameter at breast height.

(6) $$\begin{array}{c} \text{relative dominance}=\frac{\text{total basal area of the species }}{\text{total basal area of all the species}}*100. \end{array}$$

## 3. Result

### 3.1. Composition of Woody Species

**Figure 2:**
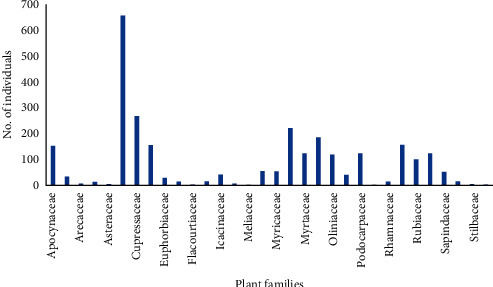
Distribution of the plant family in the Gennemar dry Afromontane forest.

A total of 55 woody species belonging to 51 genera and 34 families were recorded in the Gennemar natural forest. Approximately 22 tree species, 31 shrub species, and 2 lianas were recorded (Table 1). Fifty-three woody species were recorded inside the sample plots, while two species, *Eucalyptus globulus* and *Yushania alpina,* were recorded outside the sample plots. Celastraceae (with 659 individuals) was the most dominant family, followed by Cupressaceae (268 individuals), Myrsinaceae (222 individuals), Oleaceae (187 individuals) and Rosaceae (158 individuals). Meliaceae and Primulaceae are the least dominant plant families in the forest both with 2 individuals (Figure 2). Among the species recorded, *Jasminum stans, Maytenus addat,* and *Pittosporum abyssinicum* were endemic to Ethiopia.

### 3.2. Woody Plant Communities

**Figure 3:**
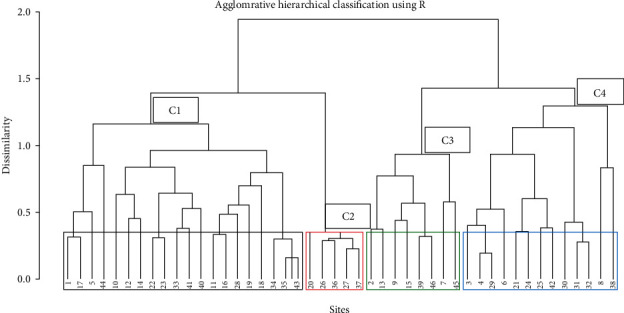
Dendrogram of the clusters (community types) of the Gennemar natural forest.

The classification of the woody vegetation of Gennemar forest resulted in four community types: *Syzygium guineense-Mystroxylon aethiopicum, Maytenus arbutifolia- Podocarpus falcatus, Myrsine Africana- Erica arborea* and *Juniperus procera-Carissa spinarum* community (Figure 3). The name for each community type was based on the importance value of the tree or shrub species. Some species were recorded in more than one community type due to low altitudinal variation in entire forest resulting occurrence of several species in wide range.

#### 3.2.1. *Syzygium guineense*-*Mystroxylon aethiopicum* Community

This community type was found between an altitudinal range of 2453–2518 m.a.s.l. and aspects of N, S, SW, NW, W, and NE. It was the most species-rich forest which consisted of 20 plots and 45 species. This community was represented by *Mystroxylon aethiopicum and Syzygium guineense.* Some species in the tree layer include *Juniperus procera, Agarista salicifolia, and Allophylus abyssinicus.* Some of the species at the shrub layer include *Asparagus africanus, Brucea antidysenterica, and Allophylus macrobotrys*. The common liana of this community was *Embelia schimperi.*

#### 3.2.2. *Maytenus arbutifolia-Podocarpus falcatus* Community

The *Maytenus arbutifolia-Podocarpus falcatus* community occurs between the altitudinal range of 2495–2562 m.a.s.l. and the aspects of SE and SW. It consisted of 5 plots and 20 species including *Maytenus arbutifolia* and *Podocarpus falcatus* as representative species. Tree species like *Mystroxylon aethiopicum, Apodytes dimidiata,* and *Bersama abyssinica* from the tree layer and *Carissa spinarum, Canthium oligocarpum,* and *Myrsine Africana* from the shrub layer were characteristic species of this community. *Embelia schimperi* and *Jasminum dichotomum* were liana species in the community.

#### 3.2.3. *Myrsine Africana-Erica arborea* Community

This community consisted of 8 plots and 33 species between altitude ranges of 2474–2517 m.a.s.l. and with aspects of N, S, W, SW, NE, and NW. *Myrsine Africana* and *Erica arborea were* representatives of this community. The tree layer consists of species such as *Juniperus procera, Bersama abyssinica,* and *Galiniera saxifraga.* The shrub layer includes *Agarista salicifolia, Allophylus macrobotrys,* and *Canthium oligocarpum.* Two liana species of this community were *Embelia schimperi* and *Jasminum dichotomum*.

#### 3.2.4. *Juniperus procera-Carissa spinarum* Community

This community consisted of 13 plots and 41 species between altitude ranges of 2505–2587 m.a.s.l. and with N, NE, NW, SE, SW, and W aspects. It is the second most species-rich community in the forest, next to the *Syzygium guineense-Mystroxylon aethiopicum community*. *Juniperus procera* and *Carissa spinarum* were the dominant species of this community. Species such as *Apodytes dimidiata, Bersama abyssinica, and Dracaena afromontana,* from the tree layer, and *Allophylus macrobotrys, Agarista salicifolia, and Canthium oligocarpum,* from the shrub layer, are dominant species of the community. *Jasminum dichotomum* was a species of liana represented in the community.

### 3.3. DBH Class Distribution

**Figure 4:**
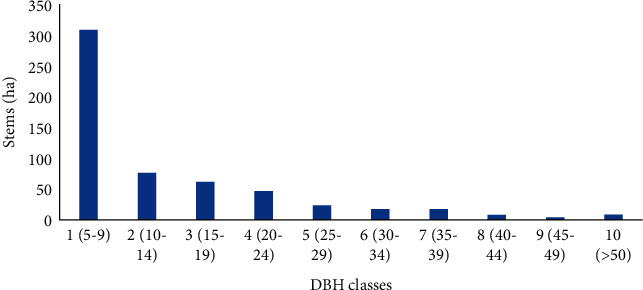
Distribution of overall woody species density among DBH classes.

According to the analysis of the distribution of DBH classes, most species had more individuals in the lower DBH classes, and it tended to decrease as DBH increased (Figure 4). This indicates that the forest has favorable regeneration potential. The highest class of DBH was recorded for *Schefflera volkensii* (130 cm), followed by *Podocarpus falcatus* and *Juniperus procera* with a DBH of 68 cm and 61 cm respectively. *Myrica salicifolia, Mystroxylon aethiopicum* and *Rhamnus staddo* occurred in lower DBH classes. *Podocarpus falcatus* and *Juniperus procera* were found in both the lower and higher DBH classes. The distribution of the DBH class shows an inverted *J-Shaped* distribution as the DBH increases, the number of individuals decreases in the higher DBH class (Figure 4). Such a pattern portrays healthy populations that are replacing themselves spontaneously through effective regeneration.

### 3.4. Basal Area Distribution

A better measure of the relative importance of tree species is provided by the Basal Area (BA) than simple stem counts [26]. As a result, the total basal area of the woody species in the forest was 19.42 m^2^·ha^−1^ (Table 2). Species with a greater DBH value contribute more to the total basal area and are considered as the most important species in the forest. *Juniperus procera* (7.79 m^2^·ha^−1^), *Podocarpus falcatus (*6.77 m^2^·ha^−1^), *Schefflera volkensii* (1.71 m^2^·ha^−1^), *and Pittosporum abyssinicum* (1.58 m^2^·ha^−1^) represented 91.84% of the total basal area. Therefore, these species are considered dominant species in the forest.

### 3.5. Importance Value Index (IVI) of Species

*Juniperus procera (*IVI = 148.5) was the most dominant and ecologically important species, followed by *Podocarpus falcatus (IVI* *=* *91.5), Maytenus arbutifolia (*IVI = 90.4)*, Mystroxylon aethiopicum ((*IVI = 69.7)*, Syzygium guineense (*IVI = 67)*, Carissa spinarum (*IVI = 64.9)*, Myrsine Africana (*IVI = 54.4)*, Jasminum stans (*IVI = 52.6)*, Erica arborea (*IVI = 51.9) and *Osyris quadripartita (*IVI = 51.5) (Table 3). Species such as *Vernonia auriculifera, Maesa lanceolata, Rhamnus prinoides,* and *Gnidia glauca* were species with a lower IVI (less than 1). Of the woody species encountered, *Jasminum stans, Maytenus addat,* and *Pittosporum abyssinicum* are endemic plants in Ethiopia (Table 4).

### 3.6. The Association of Woody Species with Altitude and Topographic Aspects

**Figure 5:**
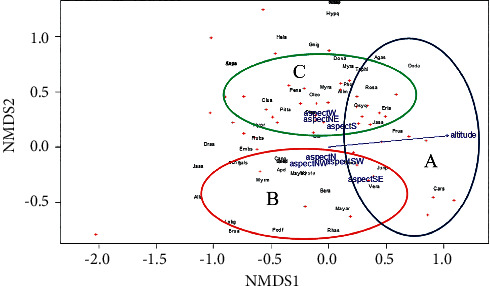
NMDS plot showing species related to altitudinal variation and topographic aspects. In both axes, cluster A shows species that are positively correlated with altitude; in the first axis, cluster B shows species that are positively correlated with topographic aspects (N, NW, SW, and SE); in the second axis, cluster C shows species that are positively correlated with topographic aspects (W, NE, and S). Abbreviated species' scientific names were described in (Table 5).

The NMDS plot shows the association of woody species to altitude and topographic aspects within the ordination of the first two axes (Stress = 0.15) (Figure 5). The species including Agas, Doda, Tephi, Rosa, Osyq, Eria, prua, Junp, Jass, Vera, and Cars are associated with altitude in the first axis. The species such as Gnig, Dova, Myrs, Phor, Myra, Allm, Pens, Clec, Clua, Pita, Rosa, Osyq, Eria, Hypr, Jass, Clua, Rhas, Podf, Lobg, Mayar, Bera, Vera, Mym, Mya, Mysta, Apd, Cano, Syzg, Schv, and Gals are associated with the NE, W, NW, SE, and SW aspects on both axes (Table 5).

The Adonis function shows that the composition varies significantly with the altitudinal gradient (Adonis, *F* (1, 38) = 3.7, P˂0.01) and the topographic aspect (*F* (6, 38) = 1.39, *P* = 0.03). The composition is more dependent on the altitudinal variation than topographic aspect.

## 4. Discussion

### 4.1. Woody Species Composition

Gennemar dry Afromontane forest is home to a diversity of 55 woody species. The number of woody species recorded in this forest is lower than in similar dry Afromontane forest types of Ethiopia. Bale mountain national park (230 species) [27], Kumuli forest (133 species) [28], Boda forest (95 species) [29], Arero forest (84 species) [30], Menagesha Suba forest (82 species) [31]. However, the number of woody species recorded was higher than in Denkoro forest (50 species) [32], Wanzaye forest (49 species) [33], Mount Duro (44 species) [34], and Chilimo forest (31 species) [35].

### 4.2. Importance Value Index

The important value index can be used to determine which species should be prioritized for conservation [36]. Higher IVIs were recorded for *Juniperus procera, Podocarpus falcatus, and Maytenus arbutifolia.* They are considered the most ecologically important woody species due to their relatively higher frequency, density, and dominance in the forest. Similar results have been reported for Juniperus procera and *Podocarpus falcatus* in Chilimo forest [37], Bale Mountains national park [27] and in Boda forest [38]. The species *Vernonia auriculifera, Maesa lanceolata, Rhamnus prinoides, and Gnidia glauca* have lower IVI and hence require conservation priority.

### 4.3. Influence of Environmental Factors

#### 4.3.1. Altitude

The plant community and species composition were influenced by altitude. Altitude is a complicated mixture of related climate variables that are strongly linked to a variety of other environmental features, such as soil texture, nutrients, and substrate stability [39]. Differences in solar radiation distribution can result in changes in microclimate (temperature) and water balance (moisture) [40]. Temperature changes over short vertical distances can be influenced by changes in altitude [41].

This has an impact on the growth, distribution, and diversity of species in forest communities [42–44]. Similarly, several studies have discovered that altitude has an important role in the distribution of woody species [45–48].

#### 4.3.2. Topographic Aspects

Topographic aspects were another determinant factor in species composition and plant community [40, 49, 50]. The differences in woody species composition across topographic aspects were most likely caused by changes in incoming solar radiation that influenced temperature and moisture. [51]. The amount of incident solar radiation can also affect the temperature of the soil and air at the ground's surface [49]. Soil temperature has a significant impact on plant growth and establishment (such as seed germination and root growth) [52]. Forest structures on northeast and south-west facing slopes are expected to differ due to differences in the duration and intensity of incoming solar radiation [40]. The northeast-facing aspects that are exposed to radiation for shorter periods of time may have a positive water balance and are more likely to maintain moisture-loving canopy trees [53]. Southwest-facing slopes get more solar radiation, both in intensity and length, than northeast-facing slopes, and are thus likely to be drier [50].

## 5. Conclusions and Recommendations

### 5.1. Conclusions

The Gennemar forest is one of the most important dry Afromontane forests in Southern Ethiopia. When compared to some of Ethiopia's dry Afromontane forests, it has a similar number of species (55 species) and more or less equal species diversity and evenness ratings. Four plant community types were identified by cluster analysis using the presence/absence value of each species in each plot. The altitude and topographic aspects determine the distributions of these woody species compositions and communities. The DBH class distribution showed that the density of individuals decreases with increasing DBH. The most economically and ecologically important woody species in the forest were *Juniperus procera and Podocarpus falcatus*. The most frequent tree species was *Mystroxylon aethiopicum*, which is similar to other dry Afromontane forests probably due to its geographic similarity to the study area.

### 5.2. Recommendations

The following recommendations are more important for the sustainability of forest biodiversity based on the findings of the current study.

- Promote community awareness campaigns about forest use through extension activities
- By discussing and consulting with local communities, strategies such as participatory forest management and community forest planting can be developed to reduce human impacts on the forest
- By implementing in situ conservation and restoration operations, the entire forest ecosystem, as well as endemic and threatened species, has to be safeguarded
- Need to take into account the determinant factors such as species type, altitude, and topographic aspect in selecting a certain forest for forestry activities
- The current study was limited to determinant factors of species composition and plant community, mainly altitude and topographic aspects; therefore, additional research is required on the soil seed bank, seed physiology, herbaceous plants, and methods of propagating ecologically and economically useful species, as well as other determinant factors of the soil

## Figures and Tables

**Table 1:** Species list recorded with Gurage local name, language, and growth habit.

Scientific name	Local name	Growth habit
*Acacia lahai*	Girar	T
*Agarista salicifolia*	Adya	S
*Allophylus abyssinicus*	Kemo	T
*Allophylus macrobotrys*	Kemo	S
*Apodytes dimidiate*	Gefea	T
*Asparagus africanus*	Yefur det	S
*Bersama abyssinica*	Hureta	T
*Brucea antidysenterica*	Abaryet	S
*Calpurina aurea*	Yefek mshra	S
*Canthium oligocarpum*	Yedbr kawa	S
*Carissa spinarum*	Wetra	S
*Clutia abyssinica*	Demesmat	S
*Dodonaea anguistifolia*	Kitkita	S
*Dovyalis abyssinica*	Koshm	S
*Dracaena afromontana*	Geshamba	T
*Ekebergia capensis*	Ner	T
*Embelia schimperi*	Qera	L
*Erica arborea*	Gedra	S
*Galiniera saxifrage*	Tikur eche	T
*Gnidia glauca*	Shera	T
*Helichrysum argyranthum*	Amedwet	S
*Hypericum quartinianum*	Abeje	S
*Hypericum revolutum*	Abeje	S
*Jasminum abyssinicum*	Abta	L
*Jasminum dichotomum*	Anfar	S
*Jasminum stans*	Bitara	S
*Juniperus procera*	Ded	T
*Lobelia giberroa*	Gimar	S
*Maesa lanceolata*	Aguaj	S
*Maytenus addat*	Shamene	T
*Maytenus arbutifolia*	Atat	S
*Myrica salicifolia*	Qechecha	T
*Myrsine Africana*	Kecho	S
*Myrsine melanophloeos*	Gomra	T
*Mystroxylon aethiopicum*	Chire	S
*Noxia congesta*	Awre	T
*Olea capensis*	Chet	T
*Olea europaea*	Weara	T
*Olinia rochetiana*	Tifeae	T
*Osyris quadripartite*	Mekekr	S
*Pentas schimperiana*	Mesafukene	S
*Phoenix reclinata*	Deye	T
*Pittosporum abyssinicum*	Emekuashe	T
*Podocarpus falcatus*	Zigba	T
*Prunus Africana*	Gereabe	T
*Rhamnus prinoides*	Geasho	S
*Rhamnus staddo*	Wach	S
*Rosa abyssinica*	Enkoche	S
*Rubus steudneri*	Ingeya	S
*Schefflera volkensii*	Angab	T
*Syzygium guineense*	Guareba	T
*Tephrosia interrupta*	Ashtatla	S
*Vernonia auriculifera*	Terashe	S

T = tree, S = shrub and L = liana.

**Table 2:** Basal area of the top 10 dominant species in the Gennemar forest.

No	Scientific name	Basal area (m^2^/ha)
1	*Juniperus procera*	7.79
2	*Podocarpus falcatus*	6.77
3	*Schefflera volkensii*	1.71
4	*Pittosporum abyssinicum*	1.58
5	*Syzygium guineense*	0.51
6	*Mystroxylon aethiopicum*	0.30
7	*Prunus africana*	0.17
8	*Myrsine melanophloeos*	0.08
9	*Apodytes dimidiata*	0.07
10	*Olea capensis*	0.06

**Table 3:** Showing the IVI value of the top 10 dominant species in the Gennemar forest.

No	Species list	Relative frequency	Relative dominance	Relative density	IVI
1	*Juniperus procera*	91.3	40.0	17.2	148.5
2	*Podocarpus falcatus*	54.3	34.8	2.3	91.5
3	*Maytenus arbutifolia*	76.1	0.1	14.2	90.4
4	*Mystroxylon aethiopicum*	67.3	1.5	0.8	69.7
5	*Syzygium guineense*	60.8	2.6	3.6	67.1
6	*Carissa spinarum*	56.5	0.0	8.4	64.9
7	*Myrsine africana*	47.8	0.0	6.6	54.4
8	*Jasminum stans*	47.8	0.0	4.8	52.6
9	*Erica arborea*	47.8	0.01	4.1	51.9
10	*Osyris quadripartita*	45.6	0.0	5.9	51.5

**Table 4:** Showing basal area of woody species in the Gennemar forest.

No.	Scientific name	Basal area (m^2^/ha)
1	*Juniperus procera*	7.79
2	*Podocarpus falcatus*	6.77
3	*Schefflera volkensii*	1.71
4	*Pittosporum abyssinicum*	1.58
5	*Syzygium guineense*	0.51
6	*Mystroxylon aethiopicum*	0.30
7	*Prunus africana*	0.17
8	*Myrsine melanophloeos*	0.08
9	*Apodytes dimidiata*	0.07
10	*Olea capensis*	0.06
11	*Olea europaea*	0.06
12	*Myrica salicifolia*	0.06
13	Dead wood	0.05
14	*Jasminum dichotomum*	0.04
15	*Allophylus abyssinicus*	0.03
16	*Bersama abyssinica*	0.03
17	*Maytenus arbutifolia*	0.02
18	*Olinia rochetiana*	0.02
19	*Maytenus addat*	0.01
20	*Brucea antidysenterica*	0.01
21	*Rhamnus staddo*	0.01
22	*Allophylus macrobotrys*	0.01
23	*Canthium oligocarpum*	0.01
24	*Hypericum revolutum*	0.01
25	*Noxia congesta*	0.01
26	*Agarista salicifolia*	0.00
27	*Erica arborea*	0.00
28	Total	19.42

**Table 5:** Abbreviations used in NMDS plotting.

Acal	*Acacia lahai*
Agas	*Agarista salicifolia*
Alla	*Allophylus abyssinicus*
Allm	*Allophylus macrobotrys*
Apd	*Apodytes dimidiata*
Aspa	*Asparagus africanus*
Bera	*Bersama abyssinica*
Brua	*Brucea antidysenterica*
Cala	*calpurina aurea*
Cano	*Canthium oligocarpum*
Cars	*Carissa spinarum*
Clua	*Clutia abyssinica*
Doda	*Dodonaea angustifolia*
Dova	*Dovyalis abyssinica*
Draa	*Dracaena afromontana*
Ekec	*Ekebergia capensis*
Embs	*Embelia schimperi*
Eria	*Erica arborea*
**G**als	*Galiniera saxifraga*
Gnig	*Gnidia glauca*
Hela	*Helichrysum argyranthum*
Hypq	*Hypericum quartinianum*
Hypr	*Hypericum revolutum*
Jasa	*Jasminum abyssinicum*
Jasd	*Jasminum dichotomum*
Jass	*Jasminum stans*
Junp	*Juniperus procera*
Lobg	*Lobelia giberroa*
Mael	*Maesa lanceolata*
Mayad	*Maytenus addat*
Mayar	*Maytenus arbutifolia*
Myra	*Myrica salicifolia*
Myrm	*Myrsine africana*
Myrs	*Myrsine melanophloeos*
Mysta	*Mystroxylon aethiopicum*
Noxc	*Noxia congesta*
Olec	*Olea capensis*
Olee	*Olea europaea*
Olir	*Olinia rochetiana*
Osyq	*Osyris quadripartita*
Pens	*Pentas schimperiana*
Phor	*Phoenix reclinata*
Pitta	*Pittosporum abyssinicum*
Podf	*Podocarpus falcatus*
Prua	*Prunus africana*
Rhap	*Rhamnus prinoides*
Rhas	*Rhamnus staddo*
Rosa	*Rosa abyssinica*
Rubs	*Rubus steudneri*
**S**chv	*Schefflera volkensii*
**S**yzg	*Syzygium guineense*
Tephi	*Tephrosia interrupta*
Vera	*Vernonia auriculifera*

## Data Availability

The data used to support the findings of this study are included within the article.
